# Use of epidemiological data as the basis for developing a medical curriculum

**DOI:** 10.1590/S1516-31802012000200007

**Published:** 2012-04-03

**Authors:** José Lúcio Martins Machado, Sonia Regina Pereira de Souza, Sylvia Michelina Fernandes Brenna, Regina Albanese Pose, Valdes Roberto Bollela, Joaquim Edson Vieira

**Affiliations:** I MD, PhD. Professor of Medicine and Dean, Universidade Cidade de São Paulo (Unicid), São Paulo, Brazil.; II MD, PhD. Assistant Professor, Universidade Cidade de São Paulo (Unicid), São Paulo, Brazil.; III MSc. Mathematician, Centro de Desenvolvimento de Educação Médica (CEDEM) “Prof. Eduardo Marcondes”, Faculdade de Medicina da Universidade de São Paulo (FMUSP), São Paulo, Brazil.; IV MD, PhD. Assistant Professor, Universidade Cidade de São Paulo (Unicid), São Paulo, Brazil.; V MD, PhD. Researcher, Centro de Desenvolvimento de Educação Médica (CEDEM) “Prof. Eduardo Marcondes”, Faculdade de Medicina da Universidade de São Paulo (FMUSP), São Paulo, Brazil.

**Keywords:** Curriculum, Epidemiology, Education, medical, undergraduate, Needs assessment, Health knowledge, attitudes, practice, Currículo, Epidemiologia, Educação de graduação em medicina, Determinação de necessidades de cuidados de saúde, Conhecimentos, atitudes e prática em saúde

## Abstract

**CONTEXT AND OBJECTIVE:**

Epidemiology may help educators to face the challenge of establishing content guidelines for the curricula in medical schools. The aim was to develop learning objectives for a medical curriculum from an epidemiology database.

**DESIGN AND SETTING:**

Descriptive study assessing morbidity and mortality data, conducted in a private university in São Paulo.

**METHODS:**

An epidemiology database was used, with mortality and morbidity recorded as summaries of deaths and the World Health Organization’s Disability-Adjusted Life Year (DALY). The scoring took into consideration probabilities for mortality and morbidity.

**RESULTS:**

The scoring presented a classification of health conditions to be used by a curriculum design committee, taking into consideration its highest and lowest quartiles, which corresponded respectively to the highest and lowest impact on morbidity and mortality. Data from three countries were used for international comparison and showed distinct results. The resulting scores indicated topics to be developed through educational taxonomy.

**CONCLUSION:**

The frequencies of the health conditions and their statistical treatment made it possible to identify topics that should be fully developed within medical education. The classification also suggested limits between topics that should be developed in depth, including knowledge and development of skills and attitudes, regarding topics that can be concisely presented at the level of knowledge.

## RESUMO

**CONTEXTO E OBJETIVO:**

A epidemiologia pode auxiliar os educadores a enfrentar o desafio de estabelecer diretrizes para o conteúdo dos currículos em escolas médicas. O objetivo foi desenvolver objetivos de aprendizagem para um currículo médico por meio de um banco de dados epidemiológicos.

**TIPO DE ESTUDO E LOCAL:**

Estudo descritivo avaliando dados de morbidade e mortalidade, realizado por universidade particular em São Paulo.

**MÉTODOS:**

O banco de dados epidemiológicos com morbidade e mortalidade registrados como resumo de causas de morte e o Disability-Adjusted Life Year (DALY) da Organização Mundial de Saúde foi utilizado. A pontuação desenvolvida considerou probabilidades de mortalidade e morbidade.

**RESULTADOS:**

A pontuação apresenta uma classificação dessas condições de saúde para ser utilizada por uma comissão responsável pelo desenho curricular, considerando seus quartis superior e inferior, designando, respectivamente, maior e menor impacto na morbidade e mortalidade. Dados de três países foram utilizados para comparação internacional, mostrando resultados distintos. As pontuações resultantes indicaram temas a serem desenvolvidos pela taxonomia educacional.

**CONCLUSÃO:**

A frequência das condições de saúde e seu tratamento estatístico possibilitaram a identificação de temas que devem ser plenamente desenvolvidos em educação médica. A classificação sugere também limites entre tópicos que devem ser desenvolvidos em profundidade, incluindo o conhecimento e o desenvolvimento de habilidades e atitudes, a respeito de tópicos que podem ser concisamente apresentados ao nível de conhecimento.

## INTRODUCTION

Recent reports have identified overall challenges and the need for innovations in the structure and process of medical education at all levels. These documents suggest that doctors need to be prepared for a more demanding society, and be ready to cope with the explosion of scientific knowledge and technology. Doctors would need to have an aptitude for lifelong learning and be prepared to be part of a working team.^1^^,^^2^^,^^3^^,^^4^

It is interesting to note that active learning principles would need to be considered as early as possible during medical school in order to provide the benefits of teamwork in areas like primary care.^5^ Nonetheless, a systematic review has suggested that active learning, like the problem-based learning approach, does not have an impact on knowledge acquisition in undergraduate medical education, even though appropriate outcome measurements need to be considered.^6^ A generalist education that could provide skills, knowledge and attitudes that make it possible to understand patients’ expectations, address wellness rather than illness alone, assimilate concepts of clinical epidemiology, develop interpersonal communication and strive to control costs would require shifts in attitude and behavior throughout the academic medical community.^7^ In Brazil, despite the large number of medical schools, just a few adopt active learning principles, and yet some reports also suggest that the traditional curriculum leaves students poorly educated about the underlying principles of the national health system.^8^^,^^9^

General medical education guidelines usually have clear statements about content and skills, but there are no priorities defining central themes. The core curriculum with special study modules was considered to be a reliable response to content overload, but there is still no suggestion about how to choose a central theme.^10^

## OBJECTIVE

The aim of this paper was to present a methodological proposal that could help curriculum managers to address this challenge, taking local and regional morbidity and mortality into consideration. We constructed an epidemiological score that can be used by specialists to develop learning objectives or even international requirements.^11^

## METHODS

Data relating to mortality (OBT) and morbidity (MRB) were taken respectively from summaries of deaths and the Disability-Adjusted Life Year (DALY) measurement of the World Health Organization (WHO) for the year of 2004.^12^ These data were chosen to present an international perspective for educational taxonomy directed by mathematical treatment.

The reported numbers of registered deaths and diseases were distributed into columns and rows according to the list provided in the WHO database. No ages or genders were identified. The probabilities were reached by means of R syntax, to reach a cumulative distribution function (CDF) for any value *x*. R is a language and an environment for statistical computing and graphics that is available as free software under the terms of the Free Software Foundation’s GNU General Public License, in source code form (http://www.r-project.org/).

The final score took into account the square root for the product of morbidity P(MRB) and mortality P(OBT) probabilities. Briefly, the frequencies were tested using the Shapiro-Wilk normality test or after Log10 transformation for both morbidity and mortality. Then, the Z numbers for both morbidity Z(MRB) and mortality Z(OBT) were obtained in order to use the definite integral, from which the results are the probability for each of the diseases from the WHO DALY and death summary lists (see Appendix for equations). In order to obtain a normal distribution, it is advisable to use a large database.

The 75^th^ percentile (quartile) from the score was used to indicate the subjects to be considered for educational taxonomy.

## RESULTS

Three countries were selected for comparison: United Kingdom (UK), Brazil (BR) and Rwanda (RW). They were representative of higher to lower-accuracy database registering, according to the WHO DALY methodology. There were 71 conditions for UK, 82 for Brazil and 87 for Rwanda that reached a score higher than zero (Table 1).

The score allowed separation of the results into quartiles. The highest quartile showed 24 health conditions for the UK, 27 for Brazil and 26 for Rwanda. Among the highest 10 health conditions for the UK, five also appeared in the Brazilian list but only one for Rwanda. On the other hand, among the 10 Brazilian health conditions, three were present in the Rwandan list (Table 2).

## DISCUSSION

This investigation used statistical data on morbidity and mortality collected by official government agencies to list and rank themes to be adopted by an undergraduate curriculum. This framework was proposed in order to manage educational taxonomic levels such as knowledge, skills and attitudes, for those in the highest quartile as well as to ensure knowledge for those in the lowest quartile.

An undergraduate curriculum should be based on and related to the needs of learners and society.^13^ However, it is not an easy task to define what a need is. Needs have a social origin and they may correspond to habits that are gradually created and also legitimized by references to ideals.^14^ Careful analysis could point out and identify needs for individuals, groups, institutions and societies. This investigation used statistical frequencies of registered health conditions as a way to identify conditions that might constitute such needs in medical education.

The taxonomy of objectives in education is a framework used to classify statements of what students are expected to learn. The domains of learning are considered to be related to cognitive, affective/attitude and psychomotor/skills. The revised Bloom taxonomy presents six major categories that differ in their complexity, such that the basic level “*remember*” is considered to be less complex than “*understand*”. From this point on, higher levels are supposed to be reached: *apply, analyze, evaluate* and *create*.^15^ Many educators have proposed that the educational process should proceed from the lowest levels to the highest levels, and also that the main goal should be the highest levels. Although the taxonomy does not propose any priority regarding the three domains (cognition, skills and attitudes), it is useful for developing educational objectives. However, one difficulty educators may have is making decisions to identify objectives between adjacent categories. Educators ought to carefully reflect on their objectives. In this regard, the taxonomies are valuable tools for defining such objectives.^16^

**Table 1.** List of conditions and scores derived from World Health Organization Disability-Adjusted Life Year (WHO DALY) database. Scores are divided by 10^5^

order	UK	Score	Brazil	Score	Rwanda	Score
1	Ischaemic heart disease	4.456	Ischaemic heart disease	2.432	Lower respiratory infections	16.410
2	Cerebrovascular disease	2.415	Cerebrovascular disease	2.205	Diarrhoeal diseases	12.090
3	Chronic obstructive pulmonary disease	1.586	Violence	2.048	HIV/AIDS	11.711
4	Trachea, bronchus, lung cancers	1.504	Lower respiratory infections	1.729	Malaria	4.742
5	Alzheimer and other dementias	1.357	Road traffic accidents	1.219	Neonatal infections and other conditions	4.694
6	Lower respiratory infections	1.119	Diabetes mellitus	1.125	Maternal conditions	3.914
7	Colon and rectum cancers	867	Chronic obstructive pulmonary disease	1.087	Tuberculosis	3.852
8	Breast cancer	783	Diarrhoeal diseases	952	Birth asphyxia and birth trauma	3.790
9	Diabetes mellitus	524	Prematurity and low birth weight	700	Prematurity and low birth weight	2.796
10	Cirrhosis of the liver	498	Hypertensive heart disease	682	Protein-energy malnutrition	1.898
11	Prostate cancer	459	Other unintentional injuries	599	Road traffic accidents	1.825
12	Lymphomas, multiple myeloma	397	Cirrhosis of the liver	549	Cerebrovascular disease	1.755
13	Oesophagus cancer	374	Endocrine disorders	546	Violence	1.544
14	Self-inflicted injuries	363	Alcohol use disorders	516	Other unintentional injuries	1.396
15	Other unintentional injuries	336	HIV/AIDS	491	Ischaemic heart disease	1.272
16	Road traffic accidents	335	Neonatal infections and other conditions	488	Meningitis	1.267
17	Pancreas cancer	317	Inflammatory heart diseases	461	Congenital anomalies	1.128
18	Alcohol use disorders	317	Breast cancer	413	Drownings	822
19	Stomach cancer	290	Protein-energy malnutrition	412	Iron-deficiency anaemia	707
20	Endocrine disorders	277	Trachea, bronchus, lung cancers	383	Diabetes mellitus	666
21	Falls	265	Congenital anomalies	378	Endocrine disorders	581
22	Asthma	253	Stomach cancer	340	Asthma	546
23	Parkinson disease	244	Tuberculosis	319	Vitamin A deficiency	488
24	Leukaemia*	239	Prostate cancer	312	Chronic obstructive pulmonary disease	483
25	Bladder cancer	233	Nephritis and nephrosis	295	Nephritis and nephrosis	445
26	Congenital anomalies	229	Falls	283	Fires*	430
27	Ovary cancer	227	Asthma*	280	Liver cancer	421
28	Drug use disorders	211	Alzheimer and other dementias	270	Syphilis	396
29	Peptic ulcer disease	190	Maternal conditions	269	Epilepsy	391
30	Inflammatory heart diseases	170	Colon and rectum cancers	269	Self-inflicted injuries	386
31	Nephritis and nephrosis	167	Self-inflicted injuries	264	War	359
32	Prematurity and low birth weight	149	Birth asphyxia and birth trauma	250	Inflammatory heart diseases	310
33	Other neoplasms	146	Drownings	220	Schistosomiasis	307
34	Hypertensive heart disease	142	Chagas disease	203	Tetanus	306
35	Melanoma and other skin cancers	136	Cervix uteri cancer	188	Hypertensive heart disease	277
36	Rheumatoid arthritis	128	Lymphomas, multiple myeloma	186	Stomach cancer	247
37	Liver cancer	127	Leukaemia	183	Cervix uteri cancer	239
38	Mouth and oropharynx cancers	122	Iron-deficiency anaemia	163	Pertussis	223
39	Epilepsy	114	Mouth and oropharynx cancers	160	Iodine deficiency	206
40	Violence	105	Meningitis	145	Measles	203
41	Cervix uteri cancer	96	Epilepsy	140	Falls	193
42	Poisonings	94	Oesophagus cancer	128	Upper respiratory infections	192
43	Skin diseases	92	Skin diseases	105	Hepatitis B	160
44	Diarrhoeal diseases	90	Pancreas cancer	97	Cirrhosis of the liver	158
45	Multiple sclerosis	90	Peptic ulcer disease	85	Peptic ulcer disease	150
46	Corpus uteri cancer	90	Ovary cancer	79	Skin diseases	149
47	Rheumatic heart disease	59	Bladder cancer	76	Lymphomas, multiple myeloma	148
48	Unipolar depressive disorders	0.2	Liver cancer	68	Trypanosomiasis	133
49	Osteoarthritis	0.1	Rheumatic heart disease	67	Alzheimer and other dementias	110
50	Birth asphyxia and birth trauma	0.05	Parkinson disease	44	Oesophagus cancer	100
51	Iron-deficiency anaemia	0.05	Other neoplasms	44	Other neoplasms	94
52	Neonatal infections and other conditions	0.05	Melanoma and other skin cancers	41	Mouth and oropharynx cancers	78
53	Schizophrenia	0.04	Unipolar depressive disorders	0.1	Rheumatic heart disease	73
54	HIV/AIDS	0.03	Drug use disorders	0.1	Hepatitis C	72
55	Fires	0.03	Schizophrenia	0.1	Poisonings	71
56	Benign prostatic hypertrophy	0.03	Rheumatoid arthritis	0.1	Colon and rectum cancers	62
57	Tuberculosis	0.03	Benign prostatic hypertrophy	0.04	Breast cancer	61
58	Meningitis	0.03	Fires	0.04	Leukaemia	58
59	Macular degeneration and other	0.02	Osteoarthritis	0.04	Pancreas cancer	49
60	Maternal conditions	0.02	Malaria	0.04	Ovary cancer	49
61	Bipolar disorder	0.02	Hepatitis C	0.04	Bladder cancer	45
62	Drownings	0.02	Schistosomiasis	0.03	Prostate cancer	38
63	Appendicitis	0.01	Corpus uteri cancer	0.03	Melanoma and other skin cancers	37
64	Hepatitis C	0.01	Hepatitis B	0.02	Trachea, bronchus, lung cancers	35
65	Upper respiratory infections	0.01	Multiple sclerosis	0.02	Schizophrenia	0.1
66	Otitis media	0.01	Bipolar disorder	0.02	Alcohol use disorders	0.1
67	War	0.005	Leishmaniasis	0.02	Osteoarthritis	0.1
68	Hepatitis B	0.004	Macular degeneration and other	0.02	Unipolar depressive disorders	0.1
69	Glaucoma	0.003	Appendicitis	0.02	Otitis media	0.04
70	Pertussis	0.002	Otitis media	0.02	Leishmaniasis	0.04
71	Periodontal disease	0.001	Upper respiratory infections	0.01	Macular degeneration and other	0.03
72			Dengue	0.01	Rheumatoid arthritis	0.03
73			Syphilis	0.01	Benign prostatic hypertrophy	0.02
74			Poisonings	0.01	Parkinson disease	0.02
75			Leprosy	0.01	Appendicitis	0.02
76			Pertussis	0.01	Gonorrhoea	0.02
77			Tetanus	0.01	Ascariasis	0.01
78			Chlamydia	0.004	Bipolar disorder	0.01
79			Ascariasis	0.003	Multiple sclerosis	0.01
80			Glaucoma	0.002	Chlamydia	0.01
81			Iodine deficiency	0.002	Diphtheria	0.01
82			Periodontal disease	0.001	Post-traumatic stress disorder	0.01
83					Drug use disorders	0.01
84					Corpus uteri cancer	0.01
85					Dental caries	0.004
86					Leprosy	0.004
87					Periodontal disease	0.003

*Lower limit of highest quartile for the country’s score.

**Table 2.** List of 10 highest scores derived from World Health Organization Disability-Adjusted Life Year (WHO DALY) database

UK	Brazil	Rwanda	Order
*Ischaemic heart disease*	*Ischaemic heart disease*	*Lower respiratory infections*	1
*Cerebrovascular disease*	*Cerebrovascular disease*	Diarrhoeal diseases	2
*Chronic obstructive pulmonary disease*	Violence	HIV/AIDS	3
Trachea, bronchus, lung cancers	*Lower respiratory infections*	Malaria	4
Alzheimer and other dementias	Road traffic accidents	Neonatal infections and other conditions	5
*Lower respiratory infections*	*Diabetes mellitus*	Maternal conditions	6
Colon and rectum cancers	*Chronic obstructive pulmonary disease*	Tuberculosis	7
Breast cancer	Diarrhoeal diseases	Birth asphyxia and birth trauma	8
*Diabetes mellitus*	Prematurity and low birth weight	Prematurity and low birth weight	9
Cirrhosis of the liver	Hypertensive heart disease	Protein-energy malnutrition	10

Underline: conditions present in both Brazil and Rwanda. *Italics:* conditions present in UK, Brazil and Rwanda.

In the medical educational field, there are curriculum proposals spanning from single themes to entire competencies.^10^^,^^17^^,^^18^ Such discussions have also been reviewed from the point of view of whether to consider that healthcare providers should have an expanded role relating to entire communities or whether the tradition of one patient’s doctor should be maintained, with regard to arranging educational priorities.^19^ However, discussion addressing the priorities for the composition of knowledge topics and skills in studying health-related issues seems to be new.

The method proposed in this study identified health conditions that could be used for construction of a medical curriculum, no matter what approach is chosen, i.e. involving either medical care providers or individual doctors, as well as in relation to any instructional design. These results established the morbidity and mortality data as reasonable sources of information for defining curriculum priorities.

Ranking of diseases according to their locally defined morbidity and mortality may suggest that some topics could be classified as presenting lower complexity in the educational taxonomy. However, this action does not eliminate the theme; rather, it instructs the learner that such knowledge could be worth remembering rather than studied up to the creative level, as would be the case for designing research proposals (higher educational taxonomic level). A recent survey of some medical schools in the United States found out that the curricula were compressed, with a large quantity of subjects, and there was no emphasis on any core competencies.^20^ The present method developed here could direct the efforts of medical educators towards how to prioritize such subjects, since there would be information to feed their reflections on the objectives.

An initiative in the United States, Undergraduate Medical Education for the 21^st^ Century (UME-21), has sponsored curricular changes focusing on core primary care clinical clerkships and outpatient settings, with an emphasis on learning objectives and competencies that would supposedly be at the center of the future healthcare system.^21^ However, the content was not taken into consideration. On the other hand, a recent study describing the process used for curriculum development stated that the disciplines chosen to run through the course were retrieved from associations of medical specialists.^22^ The results presented in this work give prominence to an epidemiological database, ensure that educational expertise from medical societies remains relevant and have the capacity to reveal the reality of local conditions.

This study has some limitations. The score was constructed using a database made available from a WHO inventory (DALY), taken from compulsory notification of diseases. Given that the data originated from government officials and agencies, some diseases with high incidence that would be considered to be public health priorities, but with low lethality and low hospital admission rates, would not be totally represented by the proposed score if they were not in the morbidity database. Proper measurement would rely more on systematic sources derived from outpatient and primary care services. Such reports would capture the spontaneous needs for healthcare, in order to constitute the proposed score. The National Ambulatory Medical Care Surveys (NAMCS) and National Hospital Ambulatory Medical Care Surveys (NHAMCS) produce annual estimates of outpatient care in the United States that show these primary diagnoses.^23^ The issue of why it would not simply be better to use prevalence to choose which diseases should be taught in undergraduate medical courses would imply accepting unreal equivalence between morbidity and mortality data. This proposed method respects both types of data, but does not emphasize either of them and allows the related database to point out their relative importance in time.

## CONCLUSIONS

In conclusion, the registered health conditions, their statistical treatment and careful analysis made it possible to identify themes that may constitute needs in medical education that have to be mastered. They also suggested that there are limits between topics that should be considered in depth at higher levels of knowledge, skills and attitudes, among those that should be worked on at lower levels of taxonomic educational complexity.
